# Experimental Investigation on the Low-Velocity Impact Response of Tandem Nomex Honeycomb Sandwich Panels

**DOI:** 10.3390/polym15020456

**Published:** 2023-01-15

**Authors:** Jinbo Fan, Penghui Li, Weiqi Guo, Xiuguo Zhao, Chen Su, Xinxi Xu

**Affiliations:** 1 Department of Medical Support Technology, Institute of Systems Engineering, Academy of Military Sciences, Tianjin 300161, China; 2 Department of Building Science, Tsinghua University, Beijing 100084, China

**Keywords:** Nomex honeycomb, polyurethane foam, hybrid core, low-velocity impact, sandwich panel, impact resistance

## Abstract

Sandwich panels are often subjected to unpredictable impacts and crashes in applications. The core type and impactor shape affect their impact response. This paper investigates the responses of five tandem Nomex honeycomb sandwich panels with different core-types under low-velocity-impact conditions with flat and hemispherical impactors. From the force response and impact displacement, gradient-tandem and foam-filled structures can improve the impact resistance of sandwich panels. Compared with the single-layer sandwich panel, the first peak of contact force of the foam-gradient-filled tandem honeycomb sandwich panels increased by 34.84%, and maximum impact displacement reduced by 50.98%. The resistance of gradient-tandem Nomex honeycomb sandwich panels under low-velocity impact outperformed uniform-tandem structures. Foam-filled structures change the impact responses of the tandem sandwich panels. Impact damage with a flat impactor was more severe than the hemispherical impactor. The experimental results are helpful in the design of tandem Nomex honeycomb sandwich panels.

## 1. Introduction

Sandwich structures typically comprise two panels and a core. The structure and material of the core influence the strength and stiffness of sandwich panels. Core structures include foam [1,2,3,4], corrugated [5,6,7], and honeycomb cores [8,9,10]. Core materials include metals [11,12,13], polymers [14,15,16], and composite materials [17,18,19,20]. Sandwich structures are widely used in aerospace and transportation fields owing to their high specific stiffness and strength [21,22,23,24].

The traditional honeycomb core has a hexagonal shape, and its mechanical properties and failure modes under different loads have been extensively investigated [25,26,27]. With the diversification of engineering applications, traditional honeycomb structures no longer meet the demands, and the optimization of honeycomb structures has become a hot research topic in various engineering fields. However, components with honeycomb sandwich structures can be subjected to unpredictable crashes and impacts in actual applications. The investigation of the low-velocity-impact response of novel structural honeycomb sandwich panels is significant.

Research on sandwich panels has reported that increasing the number of core layers can improve the impact resistance of sandwich structures [28,29,30,31,32,33]. Multi-layer sandwich panels require more material pretreatment than single-layer sandwich panels, increasing manufacturing complexity. Multi-layer honeycomb sandwich panels should also consider the misalignment of the honeycomb core layer. Although the honeycomb aperture is small enough, the misalignment caused by translation is unavoidable. The maximum contact force decreases and the crash efficiency increases as the number of layers increases for multi-layered aluminum foam cores [28,29,30]. The multi-layer sandwich panels with a corrugated core offer better impact resistance than single-layer panels, whose initial contact force increases with a decreasing depth of impact [31,32,33]. The multi-layer structure enhances the strength and energy-absorbing properties of the sandwich panels, although their mechanical behavior under out-of-plane loading varies.

In recent years, gradient strategies have become popular for sandwich structures [34,35,36,37]. Palomba et al. [34] evaluated the energy absorption of single- and double-layer aluminum honeycomb sandwich panels using low-velocity impact. The double-layer aluminum honeycomb sandwich panels exhibited high energy-absorption capabilities compared to single-layer materials. Sun et al. [34] conducted low-velocity-impact tests on homogeneous and gradient aluminum foam sandwich panels. The results showed that gradient aluminum foam sandwich panels have higher contact forces and minor indentations at low-impact energies. The gradient tandem structure can effectively improve the impact resistance of sandwich panels, which is closely related to the assembly sequence and change ratio of the gradient structure.

The core structure is a major factor affecting the strength of sandwich structures. Common methods for enhancing the strength of sandwich panels are pin-reinforced [38,39,40] and foam-filled structures [41,42,43,44]. The foam-filled honeycomb structures have better mechanical properties than the unfilled honeycomb structure. Under out-of-plane compression loading, the flexural folds of the honeycomb walls are more regular, and the wavelength of the folding wave is smaller than that of the unfilled honeycomb structure [45,46,47]. Under impact loading, the front plate deformation of sandwich panels is significantly lower [42]. Furthermore, filled foam improves the insulation and sound absorption of sandwich structures [48]. The primary factor affecting the energy absorption of foam-filled structures is the density of the foam. Nia et al. [49] investigated the effect of polyurethane foam density on the mechanical properties of aluminum honeycomb through out-of-plane quasi-static compression tests. The results indicated that the structural energy absorption increases as the density of the filled foam increases. Foam-filled lightweight polymers can significantly improve the mechanical properties of honeycomb sandwich panels, but slightly increase the total mass. Roudbeneh et al. [50] used three densities of polyurethane foam to fill a honeycomb structure. The mechanical behavior and energy absorption of the structure under impact were experimentally investigated. The results show that the honeycomb structures filled with maximum-density foam have the highest specific energy absorption. A new direction to explore is using lightweight polymer foams with different densities to fill the honeycomb structure. In summary, gradient-tandem and foam-filled structures can improve the mechanical properties of the sandwich structures.

Previous studies show that many experiments and simulations have investigated the low-velocity-impact response of single-layer honeycomb sandwich panels. Unfilled honeycombs [51,52,53,54] and polyurethane foam-filled single-layer aluminum honeycombs are the most common [55,56,57]. Increasing the number of core layers and forming the gradient structure enhance the impact resistance of sandwich panels, and filled foam is also commonly used to reinforce the performance of sandwich panels. Therefore, it is vital to understand the effect of these reinforcement methods on the impact performance of tandem Nomex honeycomb sandwich panels. However, the effect of the combination of the reinforcement methods (gradient-tandem and foam-filled) and impactor shape on tandem Nomex honeycomb sandwich panels has yet to be investigated in previous reports, and only a few studies have investigated the impact resistance of tandem honeycomb sandwich panels. This study aims to investigate the responses of five core types of tandem honeycomb sandwich panels under low-velocity impact and evaluate their impact resistance compared with that of a single-layer panel. Moreover, the effects of the core type and impactor shape are analyzed in terms of the failure mode, contact force, and impact displacement. The investigation results will help design novel lightweight sandwich panels and further explore the potential of tandem honeycomb structures.

## 2. Material and Specimen

### 2.1. Materials

**Figure 1:**
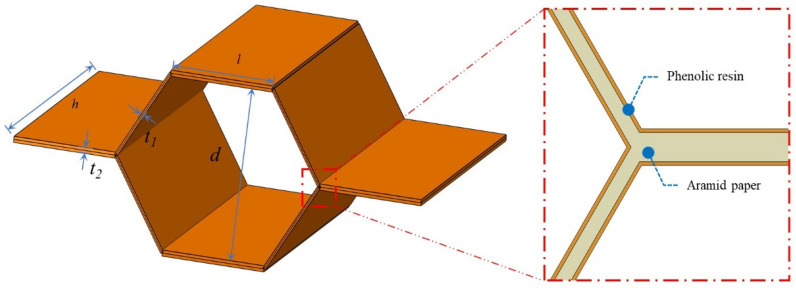
Geometry of a Nomex honeycomb cell.

The honeycomb sandwich structures consisted of four materials in this study: AL 5052-H34 (Henan Mingtai Al. Industrial Co., Ltd., Gongyi, Henan, China) as a face sheet, polyimide sheet (Suzhou Jiangnan Aerospace Mechanical & Electrical Industry Co., Ltd., Kunshan, Jiangsu, China.) as a separator, three Nomex honeycombs (PENGJI Materials Co., Ltd., Shanghai, China. equivalent density of 48 kg/m^3^ and cell lengths of 1.83, 2.75, and 3.67 mm, respectively), and three densities of polyurethane foam (Suzhou Jiangnan Aerospace Mechanical & Electrical Industry Co., Ltd., Kunshan, Jiangsu, China. body densities of 50, 60, and 80 kg/m^3^). As shown in Figure 1, the cell shape of the Nomex honeycomb was approximately hexagonal. The cell length, width, and height were defined as *l*, *d*, and *h*, respectively. The cell wall had a three-layer structure because of the manufacturing process. Nomex honeycombs were defined based on <cell length>-<height>, as summarized in Table 1. For example, “H367-8” indicates a honeycomb with a cell length and height of 3.67 and 8 mm, respectively.

### 2.2. Specimens

**Figure 2:**
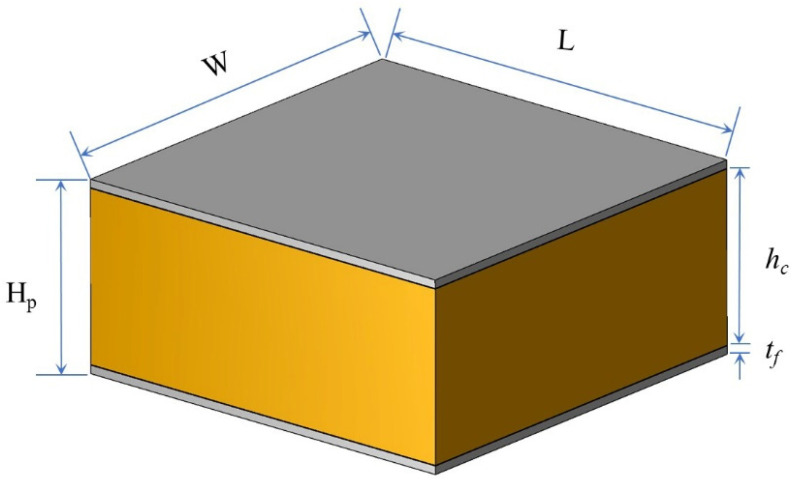
Structure of sandwich panel.

Honeycomb sandwich panels with six core types were used to investigate the low-velocity-impact response. Figure 2 shows that the sandwich panels consisted of two aluminum face sheets and a core. The dimensions of the sandwich panels were 150 mm × 150 mm × 27.4 mm (L, W, and H_p_, respectively). The face-sheet thickness was 1.2 mm, and the height of the core was 25 mm. The geometric parameters of the sandwich panels are listed in Table 2.

The core structures were divided into three types: single-layer, three-layer honeycomb, and three-layer hybrid cores. The height and cell length of the single-layer core were 25 and 2.75 mm, respectively. The three-layer honeycomb cores were composed of three Nomex honeycombs and two polyimide sheets. The three-layer hybrid core included three Nomex honeycombs filled with polyurethane foam and two polyimide sheets. The honeycomb height of the mulita-layer cores was 8 mm, and the thickness of the polyimide sheet was 0.5 mm. The face sheet, separator, and honeycomb were assembled by means of bonding using epoxy film. The sandwich panel components were bonded with epoxy film at 120 °C, using a constant pressure control to avoid adhesive spillage. The sandwich panels were pressed at 80 °C with a controlled pressure of 5 MPa. It should be noted that the core layers of honeycombs are guaranteed to be oriented in the same direction to eliminate the effect of rotation-induced misalignment in the specimens of this study. However, the influence of translation is difficult to avoid owing to the limited manufacturing process.

**Figure 3:**
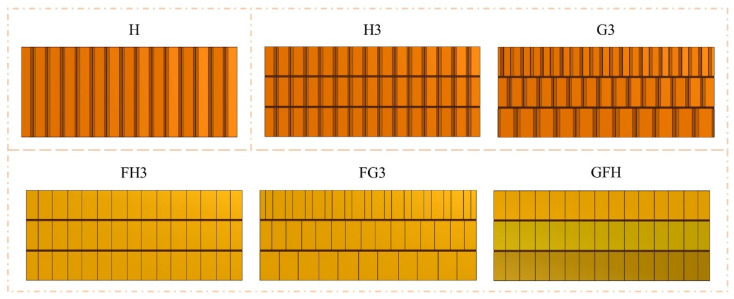
Core types of sandwich panel.

Figure 3 shows that the sandwich panels are defined based on the core types. “H,” “H3,” and “G3” represent single-layer Nomex, three-layer uniform-tandem, and three-layer gradient-tandem honeycomb sandwich panels, respectively. The foam-filled sandwich panels with uniform- and gradient-tandem structures are defined as “FH3” and “FG3”. “GFH” is gradient-filled based on FH3. The honeycomb cell length of H, H3, FH3, and GFH was 2.75 mm. The cell length of three Nomex honeycombs decreased from the bottom to top in G3 and FG3 with 3.67 mm, 2.75 mm, and 1.83 mm, respectively. In FG3 and FH3, polyurethane foam density was 50 kg/m^3^. GFH was filled with three densities of polyurethane foam, 80 kg/m^3^, 60 kg/m^3^, and 50 kg/m^3^, from the bottom up. The structure parameters of six sandwich panels are recorded in Table 3.

## 3. Experimental Methods

**Figure 4:**
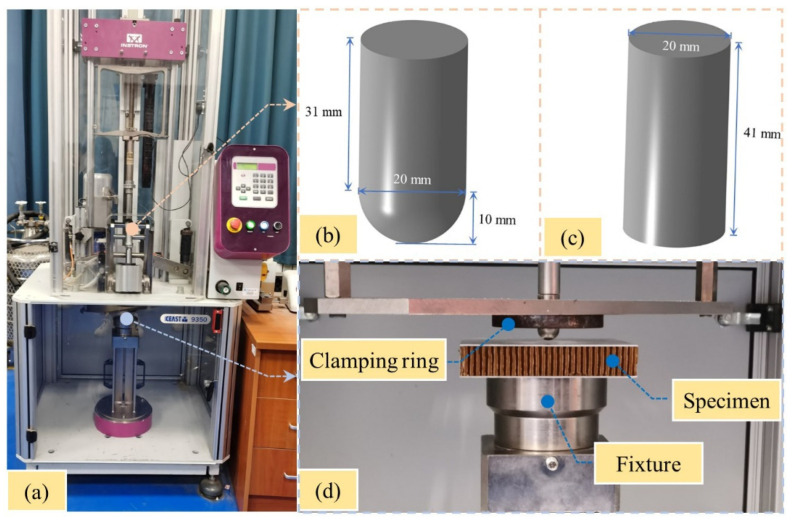
Low-velocity-impact device. (**a**) Instron CEAST 9350. (**b**) Hemisphere impactor. (**c**) Flat impactor. (**d**) Clamping system.

Low-velocity impact tests were performed on the sandwich panels using an instrumented drop-weight impact machine (Instron CEAST 9350, INSTRON Co., Canton, USA) according to ASTM D7766. As shown in Figure 4, hemispherical and flat impactors were used in the tests. The diameter and mass of the impactors were 20 mm and 5.48 kg, respectively. Two metal rings with a diameter of 76 mm were used to completely clamp the sandwich panels before the impact tests.

**Figure 5:**
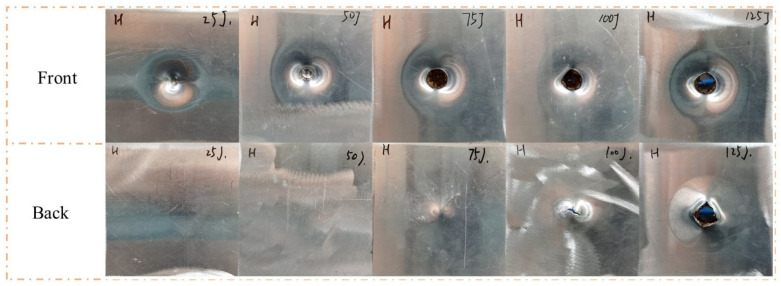
Failure modes of H under five impact energies with a hemisphere impactor.

**Figure 6:**
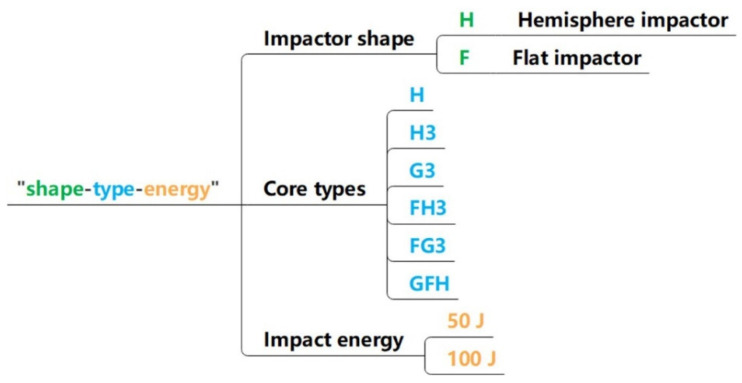
Coding for the sandwich panels.

The impact velocity depended on the impact energy and weight of the impactor. As shown in Figure 5, five low-velocity impact tests with five energies (25 J, 50 J, 75 J, 100 J, and 125 J) were conducted on H to determine the impact energies of the tandem honeycomb sandwich panel. Face sheet cracking is an essential process. Thus, the impact velocities for front and back face sheet cracking were selected as the impact velocities in this study. From the result, the impact energies are 4.27 (50 J) and 5.86 m/s (100 J). For the subsequent description, the sandwich panels were coded by the impactor shape, core type, and impact energy. As shown in Figure 6, the specimens were coded by the rule of <impactor shape>-<core type>-<impact energy>. The coding will be used for the discussion of experimental results. For example, “H-H-50” indicates that a hemispherical impactor was used to impact the single-layer honeycomb sandwich panel at 50 J impact energy.

## 4. Experimental Results and Discussion

Honeycomb sandwich panels with metal face sheets exhibit various failure modes under low-velocity impact, including the bending, indentation, cracking, and perforation of the face sheet. A damaged honeycomb contains buckling, crushing, and fracture, and a common type of damage was debonding between the interfaces of the components. The failure modes depended on the impact energy, mass, and shape of the impactor.

### 4.1. Impact Response with Hemispherical Impactor

#### 4.1.1. Failure Modes

**Figure 7:**
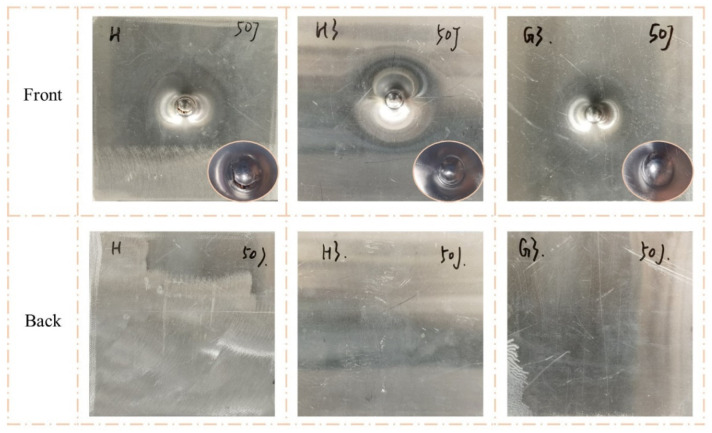
Damage modes of H, H3, and G3 under 50 J impact energy with hemisphere impactor.

**Figure 8:**
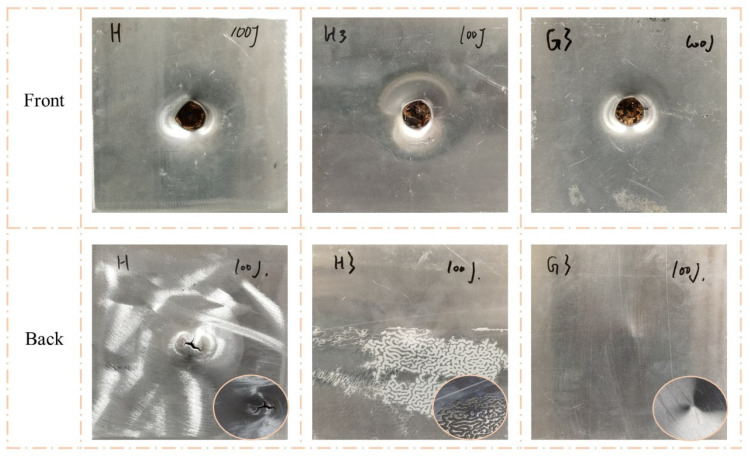
Damage modes of H, H3, and G3 under 100 J impact energy with hemisphere impactor.

Figure 7 and Figure 8 clearly show the failure modes of H, H3, and G3. When the impact energy was 50 J, the back face sheets of all three types of sandwich panels were not significantly deformed or damaged. The front face sheet of H exhibited noticeable indentation and cracking. The front face sheet of H3 had a clear indentation and minor cracking, and there was a visible indentation without cracking on G3. When the impact energy was 100 J, the front face sheets of all three types of sandwich panels exhibited perforations. However, the failure modes of the back face sheets were different. The back face sheet of H was severely deformed and exhibited extensive cracking, while there were no apparent characteristics of H3 and G3, except convex.

**Figure 9:**
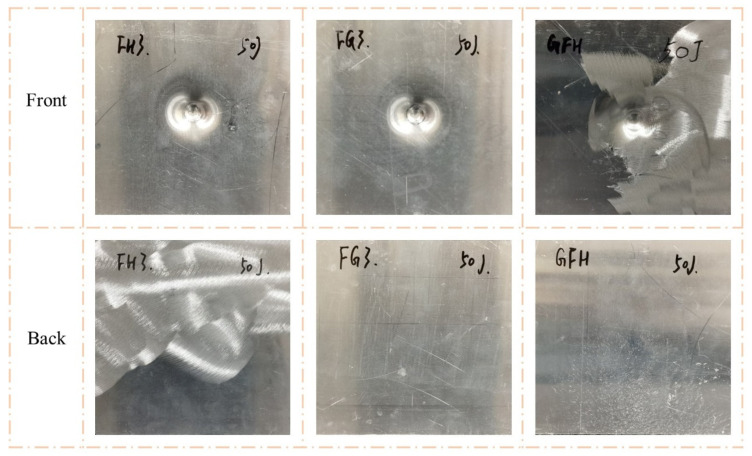
Damage modes of FH3, FG3, and GFH under 50 J impact energy with hemisphere impactor.

**Figure 10:**
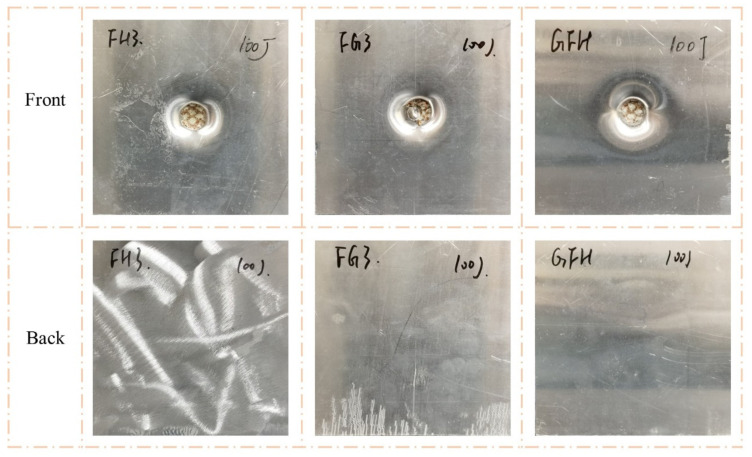
Damage modes of FH3, FG3, and GFH under 100 J impact energy with hemisphere impactor.

As shown in Figure 9 and Figure 10, the impact resistance of the sandwich panels can be effectively improved by filled the Nomex honeycomb with polyurethane foam. The visible indentations on the front face sheets of FH3, FG3, and GFH were shallower than those on G3 when the impact energy was 50 J. When the impact energy increased to 100 J, the front face sheets of FH3, FG3, and GFH formed a perforation, and the foam-filled Nomex honeycomb was apparent. Notably, the back face sheets of the three sandwich panels were almost unaffected by the two impact energies.

#### 4.1.2. Contact Force and Energy-Absorption History

**Figure 11:**
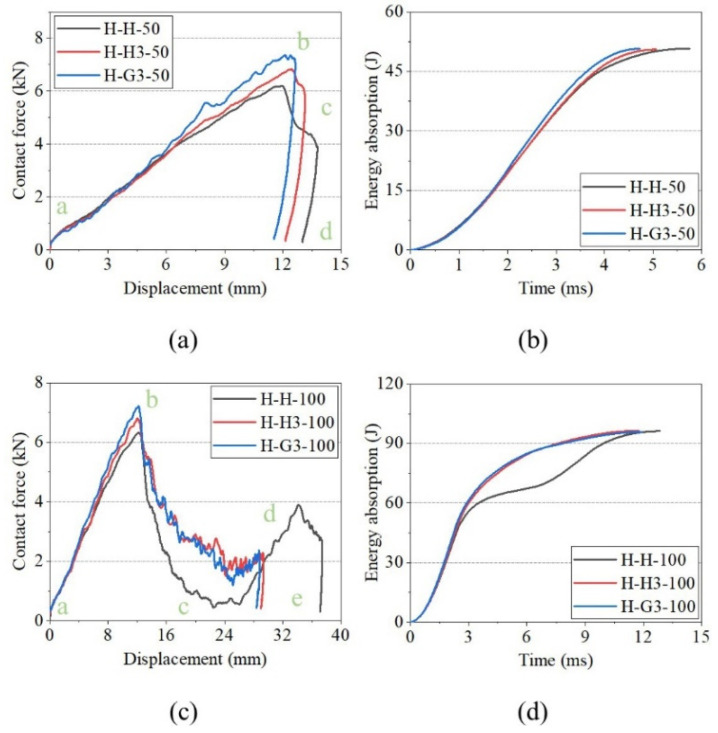
Impact response curves of H, H3, and G3 with hemisphere impactor. (**a**) Contact force–displacement curves of 50 J. (**b**) Energy-absorption curves of 50 J. (**c**) Contact force–displacement curves of 100 J. (**d**) Energy-absorption curves of 100 J.

Figure 11 shows the impact-response curves of H, H3, and G3 with the hemispherical impactor and analyses, in combination with Figure 7 and Figure 8. A linear phase (a–b) appeared before the peak of the contact force-displacement curves reached; however, it varied after the peak. At an impact energy of 50 J (Figure 11a), the contact forces of H and H3 exhibited a clear descending phase (b–c) after the peak. This indicates that the impact load on the front face sheets of H and H3 exceeded the maximum, and cracks appeared on the face sheet. The load dropped to zero (c–d), indicating that the impactor was stopped. Figure 11c shows the curves of the 100 J impact energy. The b–c stage of the curves is different, and the stage of H is lower than that of the others. Moreover, the three curves had two peaks of contact force, indicating that the impactor was crushed on the back face sheet. The curve of H had an apparent descending phase, indicating that the back face sheet was severely deformed and cracked. The energy-absorption histories of H, H3, and G3 are shown in Figure 11b,d. H3 and G3 exhibited a smoother energy-absorption history than that of H. The energy-absorption capacity of the sandwich panels was evaluated based on the damage to the sandwich panels, time of energy absorption, and smoothness of the energy-absorption history. For the same impact energy, G3 exhibited the best energy-absorption capacity, followed by H3.

**Figure 12:**
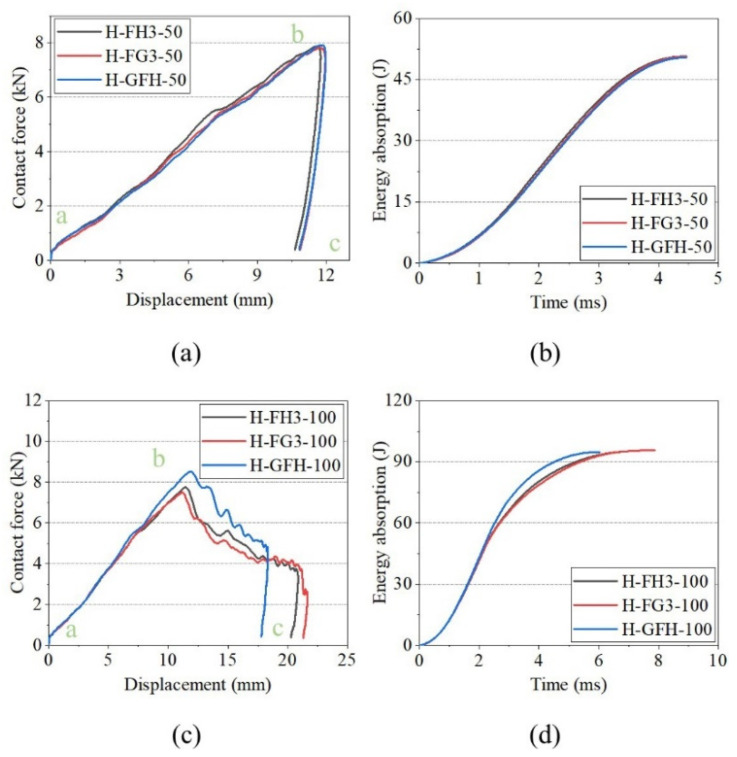
Impact responses of FH3, FG3, and GFH with hemisphere impactor. (**a**) Contact force–displacement curves of 50 J. (**b**) Energy-absorption curves of 50 J. (**c**) Contact force–displacement curves of 100 J. (**d**) Energy-absorption curves of 100 J.

Figure 12 shows the impact response curves for FH3, FG3, and GFH. Sandwich panels with different core types exhibited similar responses for the same impact energy. The curves of FH3 and FG3 almost overlapped, while GFH showed a slight difference at impact energy of 50 J. When the impact energy was 100 J, GFH had the shortest impact displacement. This indicates that the impact resistance of GFH was better than those of FH3 and FG3. The energy-absorption histories of H, H3, and G3 are shown in Figure 12b,d. The energy-absorption histories of the three sandwich panels were the same at impact energy of 50 J.

### 4.2. Impact Response with Flat Impactor

#### 4.2.1. Failure Modes

**Figure 13:**
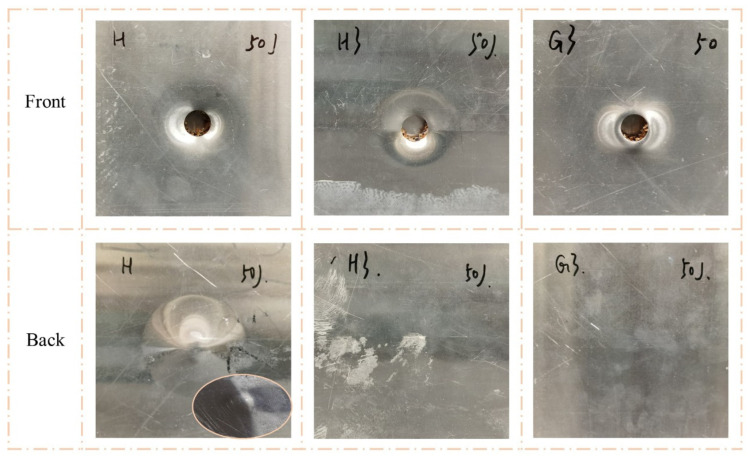
Damage modes of H, H3, and G3 under 50 J impact energy with flat impactor.

**Figure 14:**
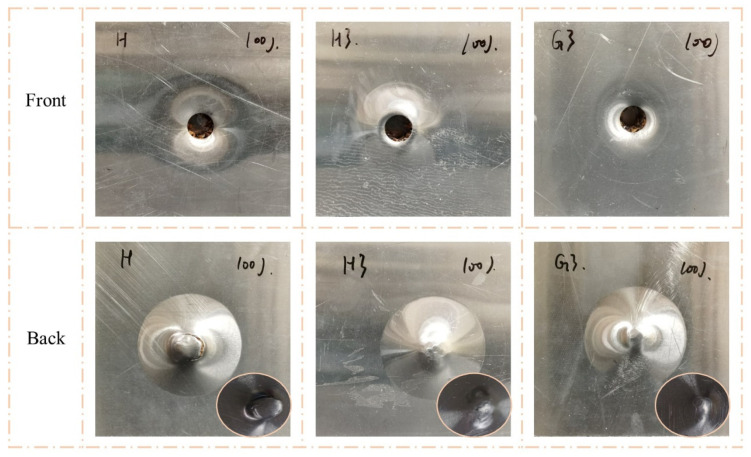
Damage modes of H, H3, and G3 under 100 J impact energy with flat impactor.

The failure modes of H, H3, and G3 under impact with a flat impactor are shown in Figure 13 and Figure 14. The front face sheet of the sandwich panels formed a perforation, and the impact caused the dislodged metal fragments to be embedded inside the honeycomb core. When the impact energy was 50 J, the back face sheet of H was severely convex, whereas H3 and G3 were flat, as before. At impact energy of 100 J, the back face sheet of H exhibited noticeable cracking, and H3 exhibited visible convexity and indentation. G3 was only convex, but had no cracking.

**Figure 15:**
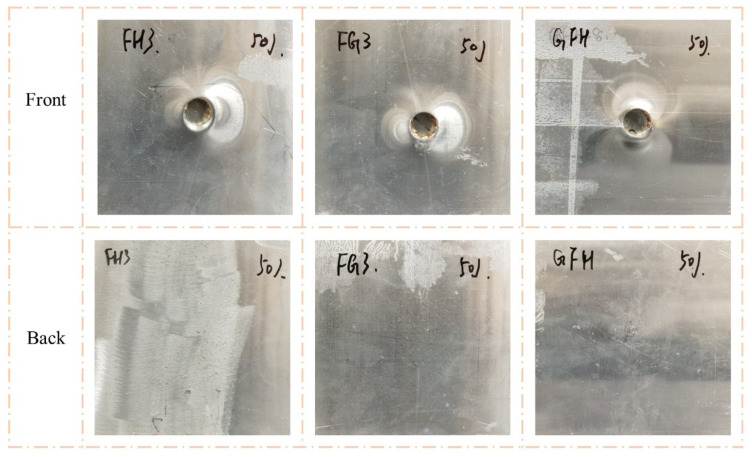
Damage modes of FH3, FG3, and GFH under 50 J impact energy with flat impactor.

**Figure 16:**
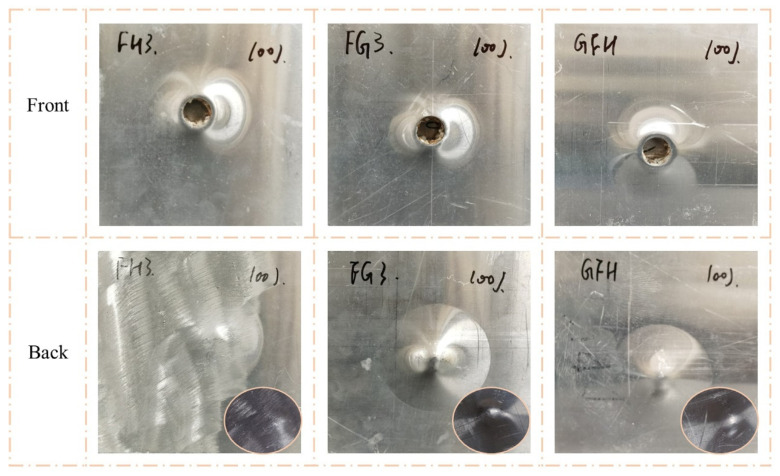
Damage modes of FH3, FG3, and GFH under 100 J impact energy with flat impactor.

Figure 15 and Figure 16 show the failure modes of FH3, FG3, and GFH under a flat impactor. The perforation of the front and convex of the back were similar for the three sandwich panels. Thus, the impact resistance of FH3, FG3, and GFH was difficult to evaluate in terms of visible damage under the same impact energy and required reference to other performances.

#### 4.2.2. Contact Force and Energy-Absorption History

**Figure 17:**
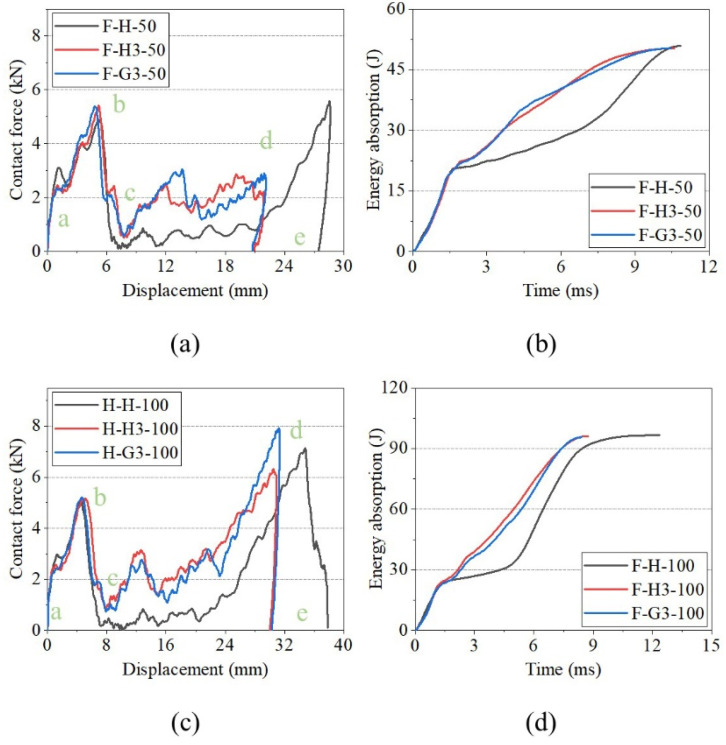
Impact responses of H, H3, and G3 with flat impactor. (**a**) Contact force–displacement curves of 50 J. (**b**) Energy-absorption curves of 50 J. (**c**) Contact force–displacement curves of 100 J. (**d**) Energy-absorption curves of 100 J.

Figure 17 shows the impact-response curves of H, H3, and G3 under a flat impactor. The contact force curves of the sandwich panels had two peaks, implying that the front face sheet had completely penetrated, and the back face sheet was crushed and deformed by the impact and metal fragment. The first and second peak forces were defined based on the contact force corresponding to the displacement position. The curves of H had the same characteristics under the two impact energies, except for the second peak force. The curves of H3 and G3 show lower peak forces in c–d, owing to the impactor penetrating the separators. When the impact energy reached 100 J, the second peak forces of H3 and G3 exceeded the first peak force. Figure 17b,d show the energy-absorption histories of H, H3, and G3. The energy-absorption capacities of H3 and G3 were better than that of H under the flat impactor.

**Figure 18:**
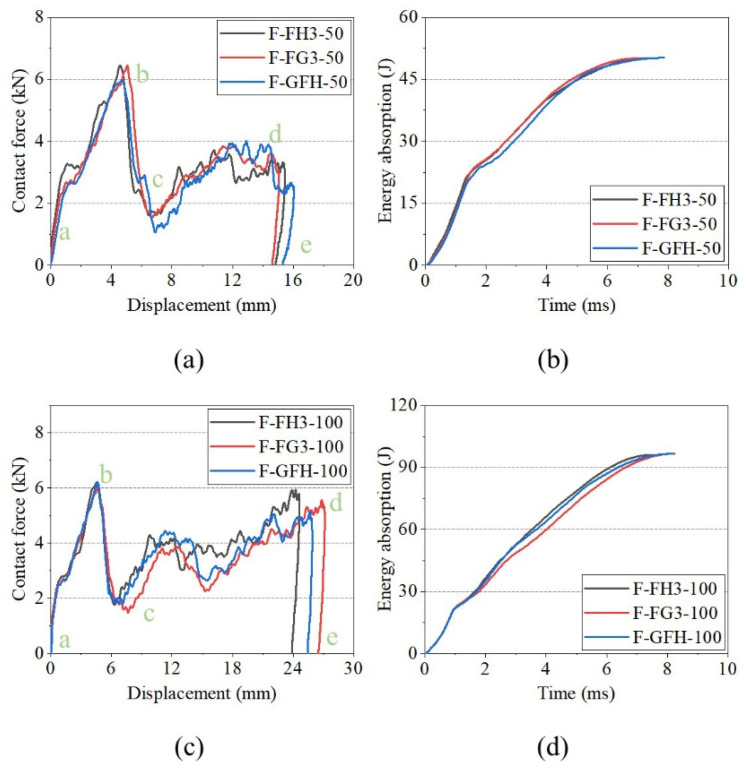
Impact responses of FH3, FG3, and GFH with flat impactor. (**a**) Contact force–displacement curves of 50 J. (**b**) Energy-absorption curves of 50 J. (**c**) Contact force–displacement curves of 100 J. (**d**) Energy-absorption curves of 100 J.

Figure 18 shows the impact-response curves for FH3, FG3, and GFH. The sandwich panels had the same first peak force under the two impact energies, and the force was greater than that of the unfilled sandwich panels. At impact energy of 50 J, there were two stages in the impact process with the flat impactor. The stage of the front face sheet penetrated (a–c), and the core crushing stage (c–e). When the impact energy was 100 J, two descending phases were observed in c–d. This indicates that the cracking damage of the separator was alleviated by the support provided by the polyurethane foam.

### 4.3. Effects of Impactor Shape on Failure Modes

**Figure 19:**
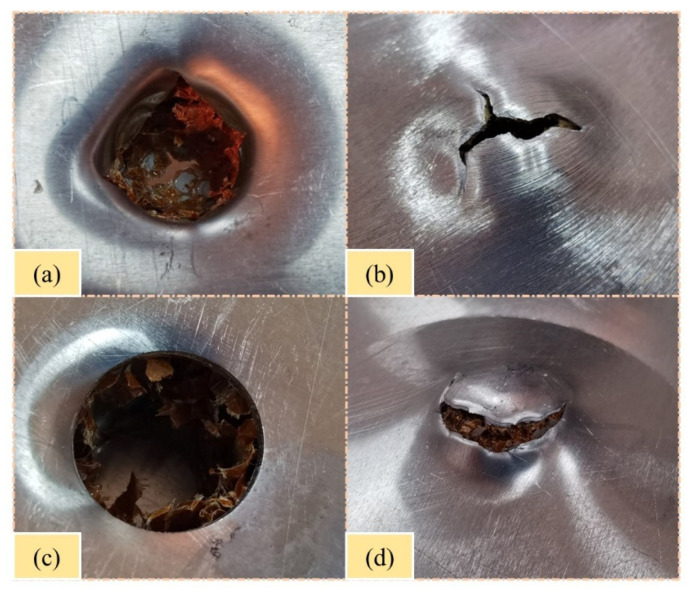
Damage morphology of H. (**a**) Front plate of H-H-100J. (**b**) Back plate of H-H-100J. (**c**) Front plate of F-H-100J. (**d**) Back plate of F-H-100J.

The effects of impactor shape were investigated by comparing the failure modes and response curves of the sandwich panels. From the results, the impact damage of the sandwich panels was closely related to the impactor shape. Figure 19 shows the damage morphology of the sandwich panels under impact energy of 100 J with hemispherical and flat impactors. The penetration shape produced by the hemispherical impactor was a concave and irregular circle with petal-like cracking. However, the penetration shape caused by the flat impactor was a convex and regular circle with smooth cracking. Furthermore, the contact force curves with flat impactors had more characteristic points and response phases. This indicates that the flat impactor produced more severe damage and complex response process than the hemispherical impactor under the same impact energy. Comparing the energy-absorption curves under the two impactor shapes, the energy-absorption curves of the sandwich panels during the impact of the hemispherical impactor were smoother than those of the flat impactor.

### 4.4. Comparison the Impact Resistance of Sandwich Panels

The impact resistance of the sandwich panels was evaluated in terms of contact force, impact displacement, and energy absorption. The sandwich panels fully absorbed 50 and 100 J of impact energy in this study. The first peak of the contact force reflects the resistance capacity of the front face sheet of the sandwich panel. Hence, the first peak force and impact displacement were regarded as indices for assessment. The change values of the impact resistance with the two impactors are summarized in Table 4 and Table 5, and the change values are based on single-layer sandwich panels.

#### 4.4.1. First Peak Force

**Figure 20:**
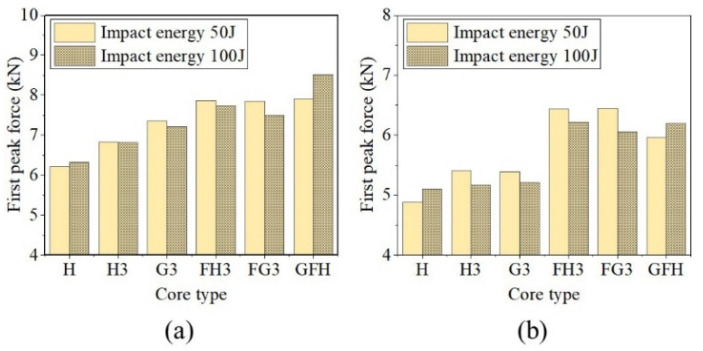
Peak force of various panels. (**a**) Hemispherical impactor. (**b**) Flat impactor.

Figure 20 shows the first peak forces of sandwich panels with different core types under hemispherical and flat impactors. Compared with the single-layer sandwich panel, tandem, gradient, and foam-filled structures effectively increased the resistance capacity of the front face sheet. For hemispherical impactors, the first peak forces of H3 under two impact energies increased by 10.07% and 7.81%, while those of G3 increased by 18.58% and 14.09%. The foam-filled structure improved the impact resistance of the sandwich panels. The first peak forces of FH3, FG3, and GFH increased by more than 18%. Under a flat impactor, the force improved to varying degrees.

#### 4.4.2. Impact Displacement

**Figure 21:**
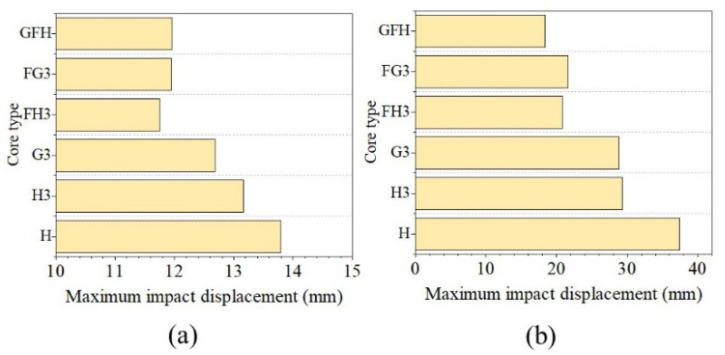
Maximum impact displacement of various panels with hemisphere impactor. (**a**) Impact energy at 50 J. (**b**) Impact energy at 100 J.

**Figure 22:**
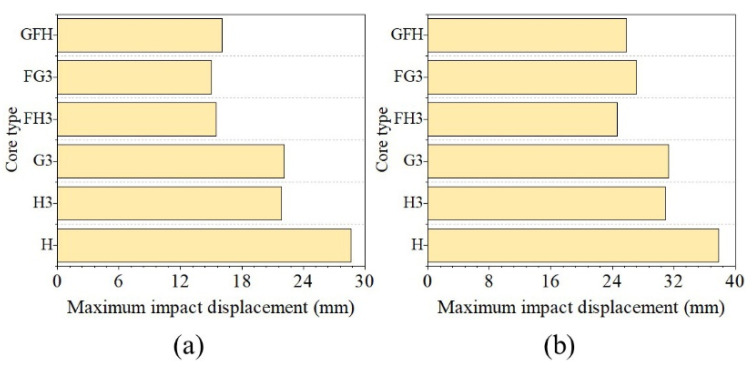
Maximum impact displacement of various panels with flat impactor. (**a**) Impact energy at 50 J. (**b**) Impact energy at 100 J.

Figure 21 shows the maximum impact displacement under the hemispherical impactor. The impact displacement of H3 decreased by 4.57% and 21.61% with 50 and 100 J impact energies and G3 decreased by 7.98% and 23%, respectively. Figure 21 shows the impact displacement of the three foam-filled sandwich panels reduced by 10% under impact energy of 50 J. For impact energy of 100 J, the impact displacement of GFH decreased by 50.98%, while those of FH3 and FG3 decreased by over 40%. However, the decreases in G3 and H3 were similar for the flat impactor as shown in Figure 22.

#### 4.4.3. Factors of Impact Resistance

By analyzing the impact resistance of H3, G3, FH3, and FG3, it was observed that foam-filled affects the impact response of the tandem honeycomb structure. G3 showed better impact resistance than H3 under two impact energies with a hemispherical impactor. However, for the foam-filled structures, the impact resistance of FH3 was better than that of FG3. Moreover, H3, G3, FH3, and FG3 exhibited sensitivity to impact energy, as 100 J impact produced a first peak force slightly less than 50 J impact energy.

The impactor shape is a significant factor in the impact response of sandwich panels. The flat impactor produces a greater impact displacement than the hemispherical impactor. From the comparison of the impact resistance of GFH under the two impactors, the sensitivity of GFH to the impact shape was observed. The flat impactor caused a significant reduction in the impact resistance of GFH.

Different shapes of impactor impact sandwich panels when the form of damage produced is different. When a hemispherical impactor impacts the sandwich panels, the contact area between the impactor and the front face sheet gradually increases. The face sheet is stretched until it is perforated and forms petal-like cracks. However, when impacting with a flat impactor, the contact area of the impactor and face sheet remains consistent. The front face sheet is sheared until it is perforated, forming rounded fragments embedded in the core and formed round cracks with neat edges.

The multi-layer sandwich panel offers a higher peak contact force than the single layer impacted with a hemispherical impactor. The failure of the multi-layer honeycomb is layer-by-layer, which can reflect the coupling effect of face sheet–honeycomb–separator. In contrast, the failure of the single-layer sandwich panel depends on the strength of the honeycomb. However, this coupling effect is inapparent with a flat impactor. In addition, G3 exhibits the highest peak contact force. It might be due to the gradient-tandem core that can better distribute the load and increase the load-bearing capacity of the front face sheet. The main form of honeycomb energy absorption forms plastic hinges, and the top core of G3 has the smallest cell length and can form the most plastic hinges. It explains that G3 has a higher peak contact force than H3 at an impact energy of 50 J.

The foam-filled structure substantially improves the peak contact force of the tandem honeycomb sandwich panel, but weakens the honeycomb-panel coupling effect. The foam retarded the formation of plastic hinges during honeycomb buckling, causing FH3 and FG3 to exhibit similar impact resistance values. The gradient-filled effect on the tandem honeycomb sandwich panels is significant by comparing the contact force-displacement curves of FG3, FH3, and GFH (Figure 12a,c).

## 5. Conclusions

The impact response of honeycomb sandwich panels with different core types of flat and hemispherical impactors was investigated using low-velocity-impact tests with impact energies of 50 and 100 J. The effects of the core type and impactor shape on the impact resistance of the honeycomb sandwich panels were analyzed by comparing them with a single-layer honeycomb sandwich panel. From the results, the following conclusions were drawn.

1. For foam-unfilled sandwich panels, the impact resistance of multi-layer honeycomb sandwich panels was better than that of single-layer honeycomb sandwich panels. The three-layer gradient-tandem honeycomb sandwich panel exhibited the best impact resistance. Compared to the single-layer sandwich panel, the first peak force increased by 18.58% and the maximum impact displacement decreased by 23%.
2. Foam-filled structures significantly improved the impact resistance of tandem honeycomb sandwich panels. When the impactor shape was hemispherical, the first peak force of a gradient-filled tandem honeycomb sandwich panel improved by 34.84%, and the impact displacement was reduced by 50.98% (compared with a single-layer honeycomb sandwich panel). However, it is sensitive to the impact shape. The change values of the first peak force were 21.97% and 21.22% under a flat impactor with 50 and 100 J of impact energy, respectively.
3. The shape of the impactor significantly affected the failure modes of the sandwich panels. The hemispherical impactor produced perforations in the shape of concave circles, forming petal-like cracking. The flat impactor caused perforation with a convex and circular hole. Furthermore, the flat impactor caused more severe damage to the sandwich panels than the hemispherical impactor with the same impact energy.
4. The foam-filled structure affects the characteristics of the impact response of the tandem honeycomb structure. Gradient-tandem honeycomb sandwich panels exhibited better impact resistance than uniform-tandem honeycomb sandwich panels. In the foam-filled structure, the two tandem structures exhibited an approximate impact resistance. However, the gradient-tandem structure was still better than the uniform-tandem structure in terms of impact displacement.
5. The low-velocity-impact response of tandem sandwich panels with different core types was investigated in this study with hemispherical and flat impactors. However, the effects caused by other shapes of impactors should have been considered. Furthermore, the effect of horizontal misalignment of the core layer of the multi-layer sandwich panel is also not considered due to the limitation of the manufacturing process.
6. When the structural parameters of gradient-tandem honeycomb sandwich panels are changed, their impact resistance also changes. In addition, the impact angle is also an influencing factor for impact damage and deserves further research. Further works are supposed to focus on developing numerical models for gradient-tandem honeycomb sandwich panels to analyze the effect of these parameters on the failure processes.

## Figures and Tables

**Table 1:** Geometrical parameters of the Nomex honeycombs.

Honeycomb Type	ρ_n_ (kg/m^3^)	c (mm)	d (mm)	h (mm)	t_1_ (mm)	t_2_ (mm)
H367-8	48	3.67	6.36	8	0.08	0.08
H275-8	48	2.75	4.76	8	0.05	0.05
H275-25	48	2.75	4.76	25	0.05	0.05
H183-8	48	1.83	3.17	8	0.05	0.05

**Table 2:** Parameters of Nomex honeycomb sandwich panel.

L (mm)	W (mm)	H_p_ (mm)	t_f_ (mm)	h_c_ (mm)
150	150	27.4	1.2	25

**Table 3:** Types of core structure.

Structure Type	Structure Name	Honeycomb Type	Foam Density
Single-layer core	H	H275-25	——
Multiple-layer core	H3	H275-8	——
	G3	H183-8 H275-8 H367-8	——
Hybrid core	FH3	H275-8	50 kg/m^3^
	FG3	H183-8 H275-8 H367-8	50 kg/m^3^
	GFH3	H275-8	50 kg/m^3^ 60 kg/m^3^ 80 kg/m^3^

**Table 4:** Change values of sandwich panels under hemispherical impactor.

Core Type	Impact Energy 50 (J)				Impact Energy 100 (J)			
	1st Peak (N)	Change Value	Disp. (mm)	Change Value	1st Peak (N)	Change Value	Disp. (mm)	Change Value
H	6205.30	0.00%	13.79	0.00%	6315.16	0.00%	37.43	0.00%
H3	6830.34	10.07%	13.16	−4.57%	6808.24	7.81%	29.34	−21.61%
G3	7358.20	18.58%	12.69	−7.98%	7204.66	14.09%	28.82	−23.00%
FH3	7855.56	26.59%	11.75	−14.79%	7741.94	22.59%	20.86	−44.27%
FG3	7837.56	26.30%	11.95	−13.34%	7490.29	18.61%	21.64	−42.19%
GFH	7909.55	27.46%	11.96	−13.27%	8515.39	34.84%	18.35	−50.98%

**Table 5:** Change values of sandwich panels under flat impactor.

Core Type	Impact Energy 50 (J)				Impact Energy 100 (J)			
	1st Peak (N)	Change Value	Disp. (mm)	Change Value	1st Peak (N)	Change Value	Disp. (mm)	Change Value
H	4884.07	0.00%	28.64	0.00%	5105.10	0.00%	37.85	0.00%
H3	5415.19	10.88%	21.9	−23.53%	5168.81	1.25%	30.91	−18.34%
G3	5385.98	10.28%	22.12	−22.77%	5214.20	2.14%	31.34	−17.20%
FH3	6437.50	31.81%	15.46	−46.02%	6217.54	21.79%	24.63	−34.93%
FG3	6445.50	31.97%	15.05	−47.45%	6051.75	18.54%	27.13	−28.32%
GFH	5957.22	21.97%	16.05	−43.96%	6188.63	21.22%	25.89	−31.60%

## Data Availability

Data will be made available on request.

## References

[1] Ivañez I., Santiuste C., Barbero E., Sanchez-Saez S. (2011). Numerical modelling of foam-cored sandwich plates under high-velocity impact. Compos. Struct..

[2] Zhou J., Hassan M.Z., Guan Z., Cantwell W.J. (2012). The low velocity impact response of foam-based sandwich panels. Compos. Sci. Technol..

[3] Kurşun A., Şenel M., Enginsoy H.M., Bayraktar E. (2016). Effect of impactor shapes on the low velocity impact damage of sandwich composite plate: Experimental study and modelling. Compos. Part B.

[4] Demircioğlu T., Balıkoğlu F., İnal O., Arslan N., Ay İ., Ataş A. (2018). Experimental investigation on low-velocity impact response of wood skinned sandwich composites with different core configurations. Mater. Today Comm..

[5] Yang X., Ma J., Shi Y., Sun Y., Yang J. (2017). Crashworthiness investigation of the bio-inspired bi-directionally corrugated core sandwich panel under quasi-static crushing load. Mater. Des..

[6] Rong Y., Liu J., Luo W., He W. (2018). Effects of geometric configurations of corrugated cores on the local impact and planar compression of sandwich panels. Compos. Part B.

[7] Wu X., Yu H., Guo L., Zhang L., Sun X., Chai Z. (2019). Experimental and numerical investigation of static and fatigue behaviors of composites honeycomb sandwich structure. Compos. Struct..

[8] Deng J., Gong X., Xue P., Yin Q., Wang X. (2022). A comprehensive analysis of damage behaviors of composite sandwich structures under localized impact. Mech. Adv. Mater. Struct..

[9] Xie S., Jing K., Zhou H., Liu X. (2020). Mechanical properties of Nomex honeycomb sandwich panels under dynamic impact. Compos. Struct..

[10] Wu J., Zhou J., Kong X., Xu Y., Chen Y., Zhu J., Jin F., Wang P. (2022). An Innovative Auxetic Honeycomb Sandwich Tube Fabrication and Mechanical Properties. Polymers.

[11] Zhang D., Fei Q., Zhang P. (2017). Drop-weight impact behavior of honeycomb sandwich panels under a spherical impactor. Compos. Struct..

[12] Uğur L., Duzcukoglu H., Sahin O.S., Akkuş H. (2017). Investigation of impact force on aluminium honeycomb structures by finite element analysis. J. Sandw. Struct. Mater..

[13] Wang Z., Wang X., Liu K., Zhang J., Lu Z. (2021). Crashworthiness index of honeycomb sandwich structures under low-speed oblique impact. Int J. Mech. Sci..

[14] Werner G., Sackman J.L. (1992). An experimental study of energy absorption in impact of sandwich plates. Int. J. Impact. Eng..

[15] Deng Y., Zhou N., Jia H., Wu H. (2022). Experimental Study on the Ballistic Resistance of S-shaped CFRP Foldcore Sandwich Structure against Flat-Nosed Projectile Impacts. Appl. Compos. Mater..

[16] Wang H., Wang W., Wang B., Fan H. (2022). Foam-filling technique to improve low-velocity impact behaviors of woven lattice truss sandwich panels. Polym. Test..

[17] Velmurugan R., Babu M.G., Gupta N.K. (2006). Projectile impact on sandwich panels. Int. J. Crashworthiness.

[18] Roy R., Park S., Kweon J., Choi J. (2014). Characterization of Nomex honeycomb core constituent material mechanical properties. Compos. Struct..

[19] Liu L., Wang H., Guan Z. (2015). Experimental and numerical study on the mechanical response of Nomex honeycomb core under transverse loading. Compos. Struct..

[20] Zhao W., Jia R., Li X., Zhao J., Xie A.Z. (2021). Flatwise compression behavior of composite Nomex (R) honeycomb sandwich structure. J. Sandw. Struct. Mater..

[21] Hong S., Pan J., Tyan T., Prasad P. (2008). Dynamic crush behaviors of aluminum honeycomb specimens under compression dominant inclined loads. Int J. Plast..

[22] Akatay A., Bora M.Ö., Çoban O., Fidan S., Tuna V. (2015). The influence of low velocity repeated impacts on residual compressive properties of honeycomb sandwich structures. Compos. Struct..

[23] Sahu S.K., Sreekanth P.S.R., Reddy S.V.K. (2022). A Brief Review on Advanced Sandwich Structures with Customized Design Core and Composite Face Sheet. Polymers.

[24] Saseendran V., Berggreen C. (2018). Mixed-mode fracture evaluation of aerospace grade honeycomb core sandwich specimens using the Double Cantilever Beam–Uneven Bending Moment test method. J. Sandw. Struct. Mater..

[25] Chen Y., Hou S., Fu K., Han X., Ye L. (2017). Low-velocity impact response of composite sandwich structures: Modelling and experiment. Compos. Struct..

[26] Dai X., Yuan T., Zu Z., Ye H., Cheng X., Yang F. (2020). Experimental investigation on the response and residual compressive property of honeycomb sandwich structures under single and repeated low velocity impacts. Mater. Today Comm..

[27] Xie S., Feng Z., Zhou H., Wang D. (2020). Three-point bending behavior of Nomex honeycomb sandwich panels: Experiment and simulation. Mech. Adv. Mater. Struct..

[28] Zhu Y., Sun Y. (2021). Low-velocity impact response of multilayer foam core sandwich panels with composite face sheets. Int. J. Mech. Sci..

[29] Wang E., Li Q., Sun G. (2020). Computational analysis and optimization of sandwich panels with homogeneous and graded foam cores for blast resistance. Thin-Walled Struct..

[30] Zhang J., Yuan H., Li J., Meng J., Huang W. (2022). Dynamic response of multilayer curved aluminum honeycomb sandwich beams under low-velocity impact. Thin-Walled Struct..

[31] Pang Y., Yan X., Qu J., Wu L. (2022). Dynamic response of polyurethane foam and fiber orthogonal corrugated sandwich structure subjected to low-velocity impact. Compos. Struct..

[32] Zou T., Tie Y., Duan Y., Cui Z., Zhan Z. (2022). Low-Velocity Impact Resistance of Double-Layer Folded Sandwich Structure. Machines.

[33] Sun Z., Shi S., Guo X., Hu X., Chen H. (2016). On compressive properties of composite sandwich structures with grid reinforced honeycomb core. Compos. Part B.

[34] Palomba G., Epasto G., Crupi V., Guglielmino E. (2018). Single and double-layer honeycomb sandwich panels under impact loading. Int. J. Impact. Eng..

[35] Sun G., Wang E., Wang H., Xiao Z., Li Q. (2018). Low-velocity impact behaviour of sandwich panels with homogeneous and stepwise graded foam cores. Mater. Des..

[36] Zhang W., Qin Q., Li K., Li J., Wang Q. (2021). Effect of stepwise gradient on dynamic failure of composite sandwich beams with metal foam core subject to low-velocity impact. Int. J. Solids Struct..

[37] Fang B., Huang W., Xu H., Jiang C., Liu J. (2022). High-velocity impact resistance of stepwise gradient sandwich beams with metal foam cores. Thin-Walled Struct..

[38] Jayaram R.S., Nagarajan V.A., Kumar K.P.V. (2017). Mechanical performance of polyester pin-reinforced foam filled honeycomb sandwich panels. Sci. Eng. Compos. Mater..

[39] Yang J., Ma L., Schröder K., Chen Y., Li S., Wu L., Schmidt R. (2018). Experimental and numerical study on the modal characteristics of hybrid carbon fiber composite foam filled corrugated sandwich cylindrical panels. Polym. Test..

[40] Taghizadeh S., Farrokhabadi A., Liaghat G., Pedram E., Malekinejad H., Mohammadi S.F., Ahmadi H. (2019). Characterization of compressive behavior of PVC foam infilled composite sandwich panels with different corrugated core shapes. Thin-Walled Struct..

[41] Burlayenko V.N., Sadowski T. (2009). Analysis of structural performance of sandwich plates with foam-filled aluminum hexagonal honeycomb core. Comp. Mater. Sci..

[42] Mozafari H., Khatami S., Molatefi H., Crupi V., Epasto G., Guglielmino E. (2016). Finite element analysis of foam-filled honeycomb structures under impact loading and crashworthiness design. Int. J. Crashworthiness.

[43] Han B., Qin K., Zhang Q., Zhang Q., Lu T.J., Lu B. (2017). Free vibration and buckling of foam-filled composite corrugated sandwich plates under thermal loading. Compos. Struct..

[44] Corigliano A., Rizzi E., Papa E. (2000). Experimental characterization and numerical simulations of a syntactic-foam glass-fibre composite sandwich. Compos. Sci. Technol..

[45] Mahmoudabadi M.Z., Sadighi M. (2011). A study on the static and dynamic loading of the foam filled metal hexagonal honeycomb—Theoretical and experimental. Mater. Sci. Eng. A.

[46] Sadowski T., Bęc J. (2011). Effective properties for sandwich plates with aluminium foil honeycomb core and polymer foam filling—Static and dynamic response. Comp. Mater. Sci..

[47] Liu Q., Fu J., Wang J., Ma J., Chen H., Li Q., Hui D. (2017). Axial and lateral crushing responses of aluminum honeycombs filled with EPP foam. Compos. Part B.

[48] Arunkumar M., Pitchaimani J., Gangadharan K., Leninbabu M. (2018). Vibro-acoustic response and sound transmission loss characteristics of truss core sandwich panel filled with foam. Aerosp. Sci. Technol..

[49] Nia A.A., Sadeghi M. (2010). The effects of foam filling on compressive response of hexagonal cell aluminum honeycombs under axial loading-experimental study. Mater. Des..

[50] Roudbeneh F.H., Liaghat G., Sabouri H., Hadavinia H. (2018). Experimental investigation of quasistatic penetration tests on honeycomb sandwich panels filled with polymer foam. Mech. Adv. Mater. Struct..

[51] Liu J., Chen W., Hao H., Wang Z. (2019). Numerical study of low-speed impact response of sandwich panel with tube filled honeycomb core. Compos. Struct..

[52] Topkaya T., Solmaz M.Y. (2018). Investigation of low velocity impact behaviors of honeycomb sandwich composites. J. Mech. Sci. Technol..

[53] Manes A., Gilioli A., Sbarufatti C., Giglio M. (2013). Experimental and numerical investigations of low velocity impact on sandwich panels. Compos. Struct..

[54] Han X., Cai H., Sun J., Wei Z., Huang Y., Wang A. (2022). Numerical Studies on Failure Mechanisms of All-Composite Sandwich Structure with Honeycomb Core under Compression and Impact Loading Conditions. Polymers.

[55] Nia A.A., Sadeghi M. (2013). An experimental investigation on the effect of strain rate on the behaviour of bare and foam-filled aluminium honeycombs. Mater. Des..

[56] Mozafari H., Molatefi H., Crupi V., Epasto G., Guglielmino E. (2014). In plane compressive response and crushing of foam filled aluminum honeycombs. J. Compos. Mater..

[57] Hussein R.D., Ruan D., Lu G., Guillow S., Yoon J.W. (2017). Crushing response of square aluminium tubes filled with polyurethane foam and aluminium honeycomb. Thin-Walled Struct..

